# Injury surveillance in community cricket: A new inning for South Africa

**DOI:** 10.4102/sajp.v78i1.1756

**Published:** 2022-06-15

**Authors:** Benita Olivier, Oluchukwu L. Obiora, Candice MacMillan, Caroline Finch

**Affiliations:** 1 Wits Cricket Research Hub for Science, Medicine and Rehabilitation, School of Therapeutic Sciences, Faculty of Health Sciences, University of the Witwatersrand, Johannesburg, South Africa; 2 Department of Physiotherapy, Faculty of Health Sciences, University of the Witwatersrand, Johannesburg, South Africa; 3 School of Medical and Health Sciences, Edith Cowan University, Joondalup, Australia

**Keywords:** cricket, sports, injury surveillance, injury prevention, injury risk reduction, data management

## Abstract

Published injury rates amongst elite and club-level youth cricketers highlight the need to implement injury risk-reducing strategies amongst the youth cricketing population. Data from sports injury surveillance systems are a prerequisite for the development and evaluation of strategies to reduce injury risk. Therefore, collecting injury surveillance data is a positive move towards reducing injuries in cricket. In South Africa, a systematic, standardised, evidence-informed injury surveillance system currently does not exist for community levels of play, namely, in cricket-playing high schools and cricket clubs. Although injury surveillance systems exist at elite levels, the obvious differences in elite versus community cricket settings mean that these systems cannot be implemented in their current form at community-level cricket. An innovative model is required to implement an injury surveillance system in community cricket.

**Clinical implications:**

This article proposes and describes a new research–practice partnership model to implement a systematic, standardised, evidence-informed injury surveillance system at cricket-playing high schools or cricket clubs within South Africa. Once this model has been employed, database systems will need to be established to allow long-term data management and sharing.

## Introduction

Epidemiological studies provide a foundational pillar for injury risk reduction and management. In cricket, as in other sports, epidemiological evidence has been used to quantify injury rates, identify underlying risk factors and describe injury severity (Ekegren, Gabbe & Finch 2016; Orchard, Kountouris & Sims 2016a). These data have informed the design and evaluation of strategies for reducing injury risk and guide the management of athletes who have already sustained such injuries. Whilst various researchers have proposed different sports injury risk reduction models (Van Dyk, Behan & Whiteley 2019), all concur that ongoing injury surveillance, as the major source of epidemiological evidence, remains essential in sport medical professionals’ quest to reduce injury incidence and severity at every level of participation.

Similar to other countries, injury surveillance databases for cricket in South Africa have been well implemented at a professional level (i.e. national team and national franchise teams). However, few standardised databases exist for the grass root levels of the sport (i.e. school or club level) (Dhillon et al. 2016; Stretch 2015). Medical professionals responsible for the respective provincial and national teams currently utilise standard injury surveillance software to capture injury data amongst professional players. However, practical and financial barriers impede the implementation of these data systems to club and school-level teams. Consequently, injury data collection of players at these levels of play has largely been restricted to independent research projects conducted by researchers at academic institutions.

Collecting data through an independent injury surveillance research project is not enough to benefit cricket. Although such research collects valuable information, it is commonly retained as standalone databases by academic institutions in a form that is not accessible to other researchers or to sports bodies to help them drive injury risk reduction. There is increasing recognition of the value of doing more with sports injury data collections by establishing data systems that will allow for effective and efficient data aggregation, management and long-term data sharing. The re-use and sharing of research data promote the dissemination of valuable information and improve the impact of research for sport. It would enable new and emerging research questions to be answered using existing data. Widespread national distribution of a proudly designed South African standardised cricket injury surveillance system amongst high school and club settings would allow more appropriate and achievable injury risk reduction and management strategies. However, in keeping with international trends, data gathered by these systems should be of such a nature that comparisons and sharing on global platforms are possible (Bahr et al. 2020).

Therefore, in this article, we highlight what is known about the injury rates in community cricket, namely amongst high school and club cricketers, why there is a need for an injury surveillance system for community level cricket, and the unique challenges likely to be faced with data collection in this context. We also propose a model called the research–practice partnership model to guide the implementation of an injury surveillance system through a meaningful collaboration for mutual benefit between a research entity and a cricket-playing high school or cricket club. We also pay attention to the notion that we need to put systems in place for the long-term management and sharing of research data to optimise their accessibility and use.

## Injury rates in cricket

As cricket is predominantly a noncontact sport, the game is generally considered a sport of low injury risk. However, repetitive strain injuries, especially amongst fast bowlers, are common, whilst projectile ball-contact injuries and fielding-related ground contact injuries also result in significant time loss amongst cricketers (Orchard et al. 2016a; Pardiwala, Rao & Varshney 2018).

Published injury rates amongst elite (Stretch 2015), club-level (Finch et al. 2010) and school-based youth cricketers (Gamage et al. 2019) highlight the need for the implementation of injury risk reduction strategies amongst the youth cricketing population. In Australia, 3.7% of children under 15 years and 7.3% of those older than 15 years who were admitted to the emergency department for a sports injury had sustained cricket-related injuries (Finch, Valuri & Ozanne-Smith 1998). Amongst sub-elite (high performing amateur) Australian club cricketers, the reported injury incidence rate was lower than at an elite level at 35.6 injuries per 10 000 playing hours and an average weekly injury prevalence of 4.1% (Soomro et al. 2018). Individual studies report injury incidence rates of 27.0% – 34.2% amongst elite youth cricketers (Milson, Barnard & Stretch 2007; Stretch 2015; Trella 2012). Amongst non-elite school-level players in South Africa (Stretch 1995), this rate was significantly higher, at 49.0%.

South Africa has been amongst the major cricket-playing countries to have published long-term elite-level injury data (Orchard et al. 2016a; Pardiwala et al. 2018). In adult club cricket, independent research projects regarding specific injury incidence and related risk factors are available (Dutton, Tam & Gray 2019; Olivier et al. 2015a, 2015b); however, studies specifically related to long-term injury incidence amongst this cohort in South Africa are scarce.

Relatively few publications have specifically described injury patterns and risk factors amongst youth cricketers (Stretch 2014). More specifically, few studies report injury incidence with large sample sizes over more than one season. Stretch (2014) investigated injury incidence amongst elite (provincial-level) schoolboy cricketers over five competitive seasons and found several differences in the nature and incidence of injury between elite adult and youth cricketers.

Whilst research regarding risk factors amongst non-elite cricket pace bowlers is available (Martin, Olivier & Benjamin 2017a, 2017b), no studies include all youth cricketers. Another limitation with existing studies is that most ignore non-time-loss injuries where players continue to play with injuries (McLeod et al. 2020; Soomro et al. 2019). Given these data gaps, it is likely that the actual injury burden amongst both youth and adult community (non-elite) level players is higher than reported, as multiple obstacles impede injury data collection (Pardiwala et al. 2018).

## Injury surveillance at a community cricket level

### Practical challenges to injury surveillance at a community level

Implementing a systematic, standardised evidence-informed injury surveillance system at a community cricket level has a number of challenges. In one study, only 23% of entities implemented a sports injury surveillance system after being introduced to it, whilst only 9% maintained the system after 2 years (Ekegren et al. 2014). Ekegren et al. (2014) found that personal (lack of importance placed on injury surveillance), sociocontextual (lack or transience of staff, under-reporting of injuries, lack of support or leadership) and systematic (time taken to upload injuries, technical issues, data requirements, adjusting to a new system) factors were barriers to the successful implementation and sustainability of an injury surveillance system.

The successful implementation of an injury surveillance system also depends on the availability of financial and operational resources (Ekegren et al. 2016) to cover the cost of purchasing software licences, technical support, ensuring compliance, education in data collection and so on. (Finch et al. 2019). In South Africa, like many other countries, community-level cricket often does not have the resources or skills to support the implementation and continued operation of an injury surveillance system. High schools and clubs do not typically have a budget for expenses such as these, although this may change in the future as they realise the value of these systems.

Capturing of injury data in community sports settings is done by volunteers, and injury surveillance is not often considered to be mandatory (Ekegren et al. 2014). Many community-level teams do not have a dedicated physiotherapist. Where an injury surveillance system is implemented, the coaching staff are expected to capture injuries as they are the closest and most consistent stakeholder involved with the team. However, capturing injury data is not part of the usual role of the coach and thus is not mandatory for them to undertake, which is a compliance barrier (Ekegren et al. 2014). In contrast, at the elite level, the motivation to comply with data-reporting requirements is often based on contractual reasons, where the capturing of injuries by the team’s sports physician or physiotherapist is part of their job description, and they identify with this role. Similar barriers have been reported in other sports and activity settings (Bromley et al. 2018; Finch et al. 2019).

These challenges to injury surveillance in community cricket can result in poor-quality data, leading to difficulty in developing injury risk reduction strategies relevant to specific target populations. The ability to evaluate the effectiveness of risk reduction strategies over time or compare injury rates amongst different sports and settings will also be compromised if the quality of the data is poor (Kucera et al. 2011). Potential sources of error in surveillance data include inconsistent application of injury definitions, incomplete data fields, misdiagnoses, missed cases and data entry mistakes (Bahr et al. 2020; Ekstrand et al. 2019). Implementing a well-described system with standardised definitions of injury is one solution to overcome some of the error sources, thereby improving the quality of data collected and eventually shared (Bahr et al. 2020; Finch & Staines 2018).

Considering these challenges, as well as the different needs of the elite setting versus the community setting, a different approach is needed towards collecting injury data in community sport (Finch 2017). To be effective in community cricket injury surveillance, systems will need to meet important characteristics described in the next section (Ekegren et al. 2016; Finch 1997; Goode et al. 2014).

### Characteristics of an effective injury surveillance system in community cricket

An effective injury surveillance system employs evidence-informed enquiry systematically in a standardised fashion (Ebrahimi et al. 2020). Developing and implementing an injury surveillance system which is informed by the evidence will increase its effectiveness. Consulting the existing research base will allow us to learn from the experiences of others. Implementing systematic processes to collect data and the standardised definitions of injury increases the quality of the data (Ebrahimi et al. 2020; Finch 1997).

An injury surveillance system for cricket must be aligned with internationally accepted standards at both the elite and community level of play. One such initiative has been the development of an international consensus statement to promote consistency in injury definitions and injury surveillance to enable the comparison of injury statistics across countries (Orchard et al. 2016b). Prior to this revised statement, injury surveillance in cricket had been conducted according to an earlier injury surveillance consensus paper (Orchard et al. 2005) in South Africa (Stretch & Raffan 2011; Stretch, Raffan & Allan 2009), Australia (Orchard, James & Portus 2006), the West Indies (Mansingh et al. 2006) and New Zealand (Frost & Chalmers 2014). Aligning an injury surveillance tool with internationally accepted standards is therefore needed to ensure that the information gained is beneficial, not only for local monitoring but also to allow for global comparisons. The injury surveillance tool now used at the elite level in South Africa is aligned with internationally accepted standards; it is now time to do the same in the case of community cricket.

Ideally, a centrally managed injury surveillance system needs to be implemented throughout South Africa, across both elite and community cricket, irrespective of the platform used to collect injury data. Whilst the type of questions asked for elite and community cricket can be as similar as the two different contexts allow, different data collection platforms may need to be used. An online injury surveillance system is found to be effective if comprehensive methods are used when collecting data (Ebrahimi et al. 2020). Ease of use of the system and the chosen platform is another facilitator of a successful injury surveillance system (Ekegren et al. 2014; Finch et al. 2019; Goode et al. 2014).

In elite or high-performance sport, injury definitions can be easier to implement, based on the professional backgrounds of aligned sports medicine professionals. At a community level, an injury definition should not depend on a healthcare professional’s diagnosis, as healthcare professionals are not widely available in this context and the system should be appropriate for use by nonhealthcare-trained staff. A consistent definition of player-reported injuries should be used to ensure that all injuries are captured (Orchard et al. 2016b) and will aid the system’s effectiveness (Ebrahimi et al. 2020). Recording both time loss and non-time-loss injuries is important because non-time-loss injuries often develop into time-loss injuries (Olivier, Lala & Gillion 2020). Limiting an injury definition only to time-loss injuries can lead to under-reporting of injuries (Orchard et al. 2016a). However, recall bias can be a common problem in sports when players are expected to recall their injuries after long periods of time (Finch 2006). A weekly questionnaire can limit this bias.

## A Research–Practice Partnership Model

**FIGURE 1:**
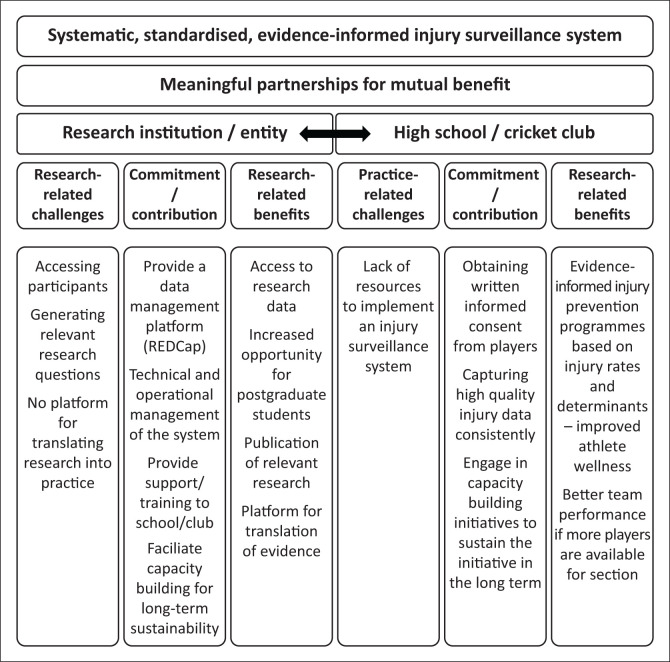
A proposed preliminary research–practice partnership model for the implementation of a systematic, standardised, evidence-informed injury surveillance system at cricket-playing high schools or cricket clubs.

We have outlined the benefits of having a systematic, standardised injury surveillance system and some of the unique challenges likely to be associated with the implementation of such a system for community cricket. Notably, the injury surveillance model employed at the elite level of cricket in South Africa does not address the needs of a community cricket setting and hence cannot be implemented. In our view, creating a meaningful partnership between a research entity and a high school or cricket club could be a way to overcome many of these challenges and to generate benefits for both, and it would ultimately allow for safe participation in community cricket and building the foundation for an active and winning nation. We propose a model for this in Figure 1.

## Challenges related to research and practice

When planning their cricket injury research, research entities can often experience challenges when it comes to identifying what the important real-life problems are, turning these into relevant research questions as well as accessing research participants. Another critical need for research entities is to have access to a platform where research findings can be applied in the sports context to create positive change. On the other hand, schools and clubs often do not have the resources to design or implement an injury surveillance system and may not even be aware of it’s benefits.

## Benefits related to research and practice

Making schools and clubs aware of the benefits of injury surveillance will increase their motivation to record injuries consistently and benefit compliance with the system. Training of coaching staff in the importance of injury surveillance and how it fits into the bigger scheme of injury risk reduction is crucial to get buy-in (Ekegren et al., 2014, Dhillon et al., 2016). Coaches’ understanding of how the injury surveillance system can benefit their professional practice, enhance players’ skills, and reduce injury can facilitate successful data collection (Ekegren et al. 2014). The research entity can offer this training through a formally recognised short course or through informal training, which routinely forms part of the research operations related to the data collection processes. As shown in other activity settings (Finch et al. 2019), online training tools can be developed and support can be provided on a personal level, when needed, to ensure that all coaches know how to use the system.

Researchers’ involvement and support for implementing injury surveillance systems and injury risk reduction programmes can facilitate adoption and maintenance of such systems (Donaldson et al. 2019; Ekegren et al. 2014; Finch et al. 2019). For example, when researchers are deployed to the study sites, their initial involvement would highlight the value of the systems to the high school or cricket club. This could lead to increased capability development of the coaching and sports administrative staff so that there would be less reliance on researchers after a number of years (Donaldson et al. 2019). Demonstrating value would encourage schools and clubs to bring their own human resources on board, such as including injury surveillance in the role description of all newly appointed coaches.

The research entities can also create sustainability of the system to ensure the system’s long-term functioning by building the capacity of researchers. This can be done by including several researchers and postgraduate students in the research unit, each researching their own unique research questions within a broader coherent research framework. Subsequently, as part of their research operations, these researchers and postgraduate students can provide direct support to the school or club to help them be motivated and commit to implementing the system and capturing the injuries. The close collaboration and increased engagement with schools and clubs may lead researchers to become aware of more relevant research questions and pursue those projects through further research. Therefore, the partnership will be a contemporary training platform for postgraduate students to do meaningful research.

Such close relationship between the school or club and researchers at the research entity will benefit both parties. The research entity will have access to research participants and their data, which will allow for enhanced opportunities to analyse and report on research findings in peer-reviewed academic journals. These publications will be disseminated amongst the academic community and form the basis of further research. Researchers are also increasingly interested in the translation of their research to directly benefit communities, and there are also opportunities for them to write papers that specifically target the sports industry and its practitioners. Ultimately, the benefits for researchers revolve around better opportunities to create new knowledge which can be practically applied within a sports setting.

Schools and clubs can benefit from the injury risk reduction benefits that an injury surveillance system brings. Teams’ performance will be enhanced if fewer players are injured, and thus more players are available for selection. Schools and clubs that implement the system will be able to use new injury data to directly inform their training programmes in collaboration with researchers and postgraduate students who work together towards evidence-based outcomes. An injury surveillance system will be an asset to schools and clubs that put a premium value on athlete health.

## Commitment and contribution

In many cases, a research institution can provide a research data management platform free of charge or at a low cost when used for research purposes. At the University of the Witwatersrand, for example, we make use of Research Electronic Data Capture (REDCap). Research Electronic Data Capture is a secure, web-based software platform designed to support data capture for studies (Harris et al. 2009, 2019). Bringing on board and training coaches on using the data reporting system can be an operation associated with the data collection processes. Not all schools and clubs have the resources to purchase a platform and support the system’s operations, but they may be willing to get on board if the necessary support is provided.

The schools and clubs will contribute to the partnership by facilitating the researchers’ access to the participants. If a school or club implements an injury surveillance system as part of its operations, cricketers can choose whether to allow their data to be used for research purposes.

## Future research directions in injury surveillance in cricket at a community level in South Africa

The research team at the Wits Cricket Research Hub for Science, Medicine and Rehabilitation has a major focus on the development and implementation of injury surveillance systems in cricket at a community level. This research team has developed the basis of a Wits Cricket Research Hub Injury Surveillance System which has been used in various longitudinal studies (Martin et al. 2017b; Olivier et al. 2017, 2020). The system must be adapted to meet the needs of a community cricket setting before widespread implementation. A first step in establishing an injury surveillance system is to scope the evidence about how injury surveillance is undertaken across the world and in other sports. The aim is not to purely adopt systems used elsewhere but to learn from others and adapt what we have learnt to our own unique South African context. Determining the current injury surveillance methods used at cricket-playing schools and cricket clubs internationally is a crucial step in tailoring an injury surveillance system specifically for South African community cricket. Such information includes the platforms used to capture data, implementation processes and methods in which compliance and data accuracy are optimised, as well as the perceptions, barriers and facilitators to the implementation of such systems. We intend to explore these factors in our own sporting context through interviews and collaboration with key stakeholders to determine their perceptions towards injury surveillance. We will use principles of implementation science (Donaldson & Finch 2012; Finch 2011) and will consider the stages of community readiness, the implementation drivers and the stages of the implementation process (Fixsen et al. 2005) appropriate to the sports injury risk-reduction field (Finch & Donaldson 2010).

Ensuring content validity and acceptable test-retest reliability of the injury surveillance tool within our local context will also be important. Survey questions will need to be worded appropriately to ensure adequate understanding, for example, when used in younger populations such as high school and primary school players. Audits of the data quality are essential (Ekegren et al. 2016). Ongoing monitoring and evaluating of the system’s implementation, barriers to its use and the quality of the output will be essential to refine and improve the injury surveillance system continuously.

After implementing the Wits Cricket Research Hub Injury Surveillance System in a number of cricket-playing high schools and cricket clubs, the Reach-Efficacy-Adoption-Implementation-Maintenance (RE-Aim) framework will be used to evaluate the success of its implementation, similar to Ekegren et al. (2014). Reach will be defined as the proportion of the target population of cricket-playing schools and cricket clubs that have been informed or trained on the use of the Wits Cricket Research Hub Injury Surveillance System. Efficacy will relate to the quality of the data collected, including its validity and completeness. Adoption will be assessed as the proportion of high schools or cricket clubs that agree to participate in the injury surveillance initiative and implementation as the proportion of high schools or clubs which actively record injuries during the season using the Wits Cricket Research Hub Injury Surveillance System. Maintenance will be defined as the extent to which the continuous capturing of injuries is sustained over consecutive seasons.

Once the system is implemented and tested over a few seasons, injury epidemiology will be determined according to the ‘translating research into injury prevention practice’ framework (Finch 2006), where injury data will inform other injury-risk reduction strategies. Furthermore, we will refine the research–practice partnership model further by informing it with more evidence and learning from our empirical experiences.

## Injury surveillance research data management and sharing

Ongoing injury surveillance will enable many research questions to be answered, policy to be informed, change to be effected in the long term and new and emerging issues identified proactively. Irrespective of whether injury data are being collected through formal, ongoing injury surveillance systems or independent research projects, processes need to be in place to allow responsible long-term curation, reuse and sharing of research data.

Dealing with cricket research data has challenges that include making a social, cultural and technological shift that would enable data sharing. The *Protection of Personal Information Act* which came into effect on 01 July 2021 in South Africa (Malherbe 2021) for data management and storage, controlled access and scientific integrity requires consideration for long-term data curation and sharing (Meyer 2018).

Improved knowledge about research data management and sharing of cricket injury surveillance across South Africa will encourage collaborative projects, formulation of new research questions and avoid unnecessary and unintentional duplication of research resources (Pisani et al. 2010), thereby saving scarce resources (Obiora et al. 2021). We will need a strategic approach to guide data collection and management to enable the combination of various datasets, allowing advanced data analysis (Big Data) in the future. Merging smaller datasets into a larger dataset will have more statistical power, yield more robust conclusions and recommendations and increase the confidence of the cricket community to be able to respond to the results and ultimately implement strategies to minimise injuries.

## Conclusion

Implementing a systematic, standardised, evidence-informed injury surveillance system is the first building block of any injury-risk reduction strategy. The proposed research–practice partnership model would meet the needs of research entities as well as high schools and cricket clubs. It is essential to collect injury surveillance data and curate it responsibly to allow researchers to create an impact on cricket. Research data curated responsibly will go a long way to answer contextually relevant questions to enhance performance and reduce injury risk.
