# Erratum: Injury surveillance in community cricket: A new innings for South Africa

**DOI:** 10.4102/sajp.v78i1.1815

**Published:** 2022-08-31

**Authors:** Benita Olivier, Oluchukwu L. Obiora, Candice MacMillan, Caroline Finch

**Affiliations:** 1 Wits Cricket Research Hub for Science, Medicine and Rehabilitation, School of Therapeutic Sciences, Faculty of Health Sciences, University of the Witwatersrand, Johannesburg, South Africa; 2 Department of Physiotherapy, Faculty of Health Sciences, University of the Witwatersrand, Johannesburg, South Africa; 3 School of Medical and Health Sciences, Edith Cowan University, Joondalup, Australia

In the original published article, Olivier, B., Obiora, O.L., MacMillan, C. & Finch, C., 2022, ‘Injury surveillance in community cricket: A new inning for South Africa’, *South African Journal of Physiotherapy* 78(1), a1756. https://doi.org/10.4102/sajp.v78i1.1756, the title contained a spelling error. The spelling error was unintentional. As a result, the title is corrected to read ‘Injury surveillance in community cricket: A new innings for South Africa’.

The publisher apologises for this error. The correction does not change the study’s findings of significance or overall interpretation of the study’s results or the scientific conclusions of the article in any way.

